# Surgical outcome of laminoplasty for cervical spondylotic myelopathy: a single-institution experience

**DOI:** 10.3906/sag-2102-308

**Published:** 2021-04-21

**Authors:** M. Erhan TÜRKOĞLU, Ahmet GÜLMEZ, Çağirı ELBİR, Ömer Selçuk ŞAHİN, Şahin HANALİOĞLU, Samet DİNÇ, Çağhan TÖNGE, Mehmet KALAN

**Affiliations:** 1 Department of Neurosurgery, Dışkapı Yıldırım Beyazıt Education and Research Hospital, Faculty of Gülhane Medicine, University of Health Sciences, Ankara, Turkey; 2 Neurosurgery Clinic, Yozgat City Hospital, Ministry of Health, Yozgat, Turkey; 3 Department of Neurosurgery, Mengücek Gazi Education and Research Hospital, Erzincan Binali Yıldırım University, Erzincan, Turkey; 4 Department of Neurosurgery, Faculty of Medicine, Hacettepe University, Ankara, Turkey

**Keywords:** Cervical spondylotic myelopathy, K-line, laminoplasty, sagittal balance

## Abstract

**Background/aim:**

Cervical spondylotic myelopathy (CSM) develops as a result of compression of the spinal cord in the cervical region. Early diagnosis and surgical treatment can limit the progression of symptoms. Various surgical approaches and strategies have been described in the literature. This study aims to evaluate the clinical and radiological results of open-door laminoplasty for the treatment of CSM.

**Materials and methods:**

In this study, we retrospectively analyzed the patients who underwent expansive open-door laminoplasty secured with titanium miniplates. Thirty-four patients with CSM who were followed up postoperatively for more than 12 months were included in the study. The modified Japanese Orthopaedic Association (mJOA) score was used to assess the degree of myelopathy. We evaluated cervical sagittal alignment with C2–C7 Cobb angle, the ambulatory status with the Nurick grade, and measured postoperative neck pain with the visual analogue scale (VAS).

**Results:**

Themeanm JOA score was 11 (range 6–15) preoperatively, and 13.5 (range 9–16) postoperatively with an average 55% recovery rate (range 0–75) (p < 0.001). Themean–Nurick grade was 2 (range 1–3) preoperatively and 1 (range 0–3) postoperatively (p < 0.001). The median cervical lordotic angle increased from 7.5 ° preoperatively to 12.5 ° postoperatively (p = 0.044). K-line (+) patients› mean mJOA scores significantly increased from 10.8 ± 1.7 to 13.3 ± 1.7 postoperatively (p < 0.001). The mean preoperative VAS reduced from 2.66 ± 1.4 to 1.59 ± 1.4 postoperatively (p < 0.001).

**Conclusion:**

Open-door laminoplasty technique is an effective surgical procedure that can be used safely to treat cervical spondylotic myelopathy. Our findings suggest that it can limit the progression of symptoms and alter the poor prognosis in CSM.

## 1. Introduction

Cervical spondylotic myelopathy (CSM) occurs as a result of spinal cord compression from anterior or posterior due to spinal degeneration orspondylosis, in which adjacent vertebrae are involved [1,2]. It is most commonly seen in elderly patients due to intervertebral disc degeneration, ligamentum flavum hypertrophy, facet, or uncovered tebral join to osteophytes [1,3]. It causes functional disability and a decline in the performance of daily activities. Most patients suffer from gradual, stepwise deterioration without spontaneous resolution [4]. Understanding the clinical details and the pathophysiology of CSM is essential because early surgical treatment can limit the progression of symptoms, improve prognosis, and change the natural history of CSM [5,6]. Laminoplasty for CSM is a commonly used technique worldwide and allows the decompression of multi-level pathology without fusion, preserving the cervical spine’s motion, and can also be combined with the anterior approach [1,2]. In properly selected patients, the results of laminoplasty operation are satisfactory, with fewer complications and more favorable results compared with the laminectomy procedure [7,8]. Even in the elderly with various medical conditions, laminoplasty has better surgical outcomes [6,9,10].

Our study aims to evaluate the clinical and radiological results of CSM patients who underwent expansive open-door laminoplasty secured with miniplates at our institution.

## 2. Materials and methods

This is a single-center, retrospective cohort study and ethics approval of the study was obtained from the Ethics Committee for Clinical Studies at the University of Health Sciences Diskapi Yildirim Beyazit Training and Research Hospital (Date: February 22, 2021; Number: 105/18).

### 2.1. Patient population

In this study, 34 consecutive patients who were operated for CSM between April 2015 and December 2019 at the Neurosurgery Clinic of University of Health Sciences Diskapi Yildirim Beyazit Training and Research Hospital were included. Table 1 is a demonstrated summary of demographic and clinical characteristics of the patients (Table 1). The patients’ median age was 63.7 ± 10.3 years (range 33–80 years). The male/female ratio was 2.4. All patients had at least 12 months of follow-up period after the operation (mean 12.6 ± 2.3, range 2–26 months). Patients with K-line (−) ossification of the posterior longitudinal ligament (OPLL), spinal malignancy and infection, and patients with cervical spinal trauma were not included in the study.

### 2.2. Surgical technique

The patients were positioned prone, and the head slightly flexed to enlarge the spinal canal. Patients were intubated with a fiberoptic or video laryngoscope to avoid extreme neck hyperextension. During the surgical procedure, the patients’ neurological status was monitored using motor evoked potentials (MEPs) and somatosensory evoked potentials (SSEPs). Open door laminoplasty was performed according to the technique described by Kurokawa et al. [11]. Midline skin incision, subperiosteal muscle dissection toward the lamina-lateral mass junction with electrocautery were performed. First of all, we performed the opening gutter on the symptomatic side, which required for aminotomies due to myeloradiculopathy. Then, we created the gutter with a high-speed drill (NSK, Nakanishi Inc. 700 Shimohinata Kanuma-shi Tochigi, Japan) on the lamina and lateral mass junction.

**Figure 1A:**
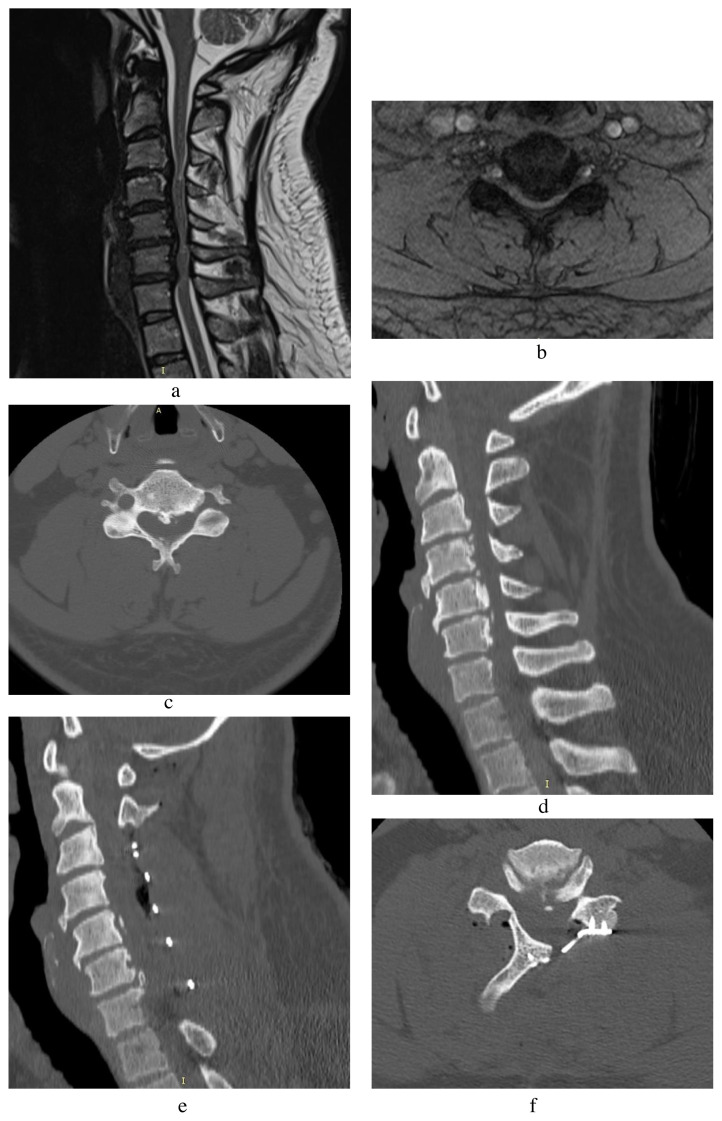
Sagittal T2-weighted MRI of a patient is demonstrating severe cervical spinal canal stenosis from C3 to C7, especially C3–4 and C5–6 due to degenerative disc protrusions and narrow spinal canal, causes complete CSF erasure and spinal cord compression at these levels. There is a high T2 signal noted within the cord due to both edema and myelomalacia. **B**. Axial T2-weighted MRI at C5–6 level is demonstrating severe spinal canal stenosis. **C**. Axial CT at C5–6 level is demonstrating intense calcification and spinal cord compression. **D**. Preoperative sagittal CT is demonstrating dense osteophyte formation which is apparent in both anterior and posteriorly. **E**. Postoperative sagittal CT is showing enlargement and space gained in the spinal canal. **F**. Postoperative axial CT is showing open door laminoplasty with titanium screws and plate and enlargement of the spinal canal.

We drilled each level, both outer cortex, and medullary bone. We observed the yellowish ligamentum flavum and then the bluish dural sac through the inner cortex’s remaining part. We resected the inner cortex with a Kerrison rongeur after the removal of an appropriate amount of bone. The lamina’s cranial side is covered or overlapped by the caudal lamina of upper vertebra and was thicker than the caudal side, and has no ligamentum flavum. Therefore, we paid great attention during the cranial dissection and removal of the cranial side. Then, via the help of a high-speed drill, a hinge gutter was created at the intersection of the opposite lamina and facet joints, provided that the inner cortex was preserved. After both open side and hinge side gutters had been performed, the lamina was manipulated dorsolaterally like opening a door, and the underlying ligamentum flavum resected with a Kerrisonrongeur with an introduced tension along with dorsolateral manipulation. After the elevation of lamina, we controlled venous bleeding from epidural space with SURGICEL (Johnson & Johnson Medical Devices Companies, Ethicon, NJ, USA), bipolar coagulation, or a combination of both. As a final step, segmental fixation with titanium miniplate-screw (Osimplant, Ikitelli, İstanbul, Turkey) system was performed unilaterally to maintain the expansion of the spinal canal in an open-door fashion. We placed two screws on the lamina’s free edge and the medial facet, respectively (Figures 1A–F).

### 2.3. Postoperative care

All patients were mobilized on the first operative day. We ordered a cervical collar for the patients at least 3 weeks after surgery. An early rehabilitation program was performed if necessary.

### 2.4. Clinical evaluation and follow-up

Modified Japanese Orthopaedic Association (mJOA) score was used to evaluate myelopathy’s degree both preoperatively and postoperatively. Our team did postoperative evaluations 12 months after the initial surgery. JOA score recovery rate, which indicates the postoperative improvement, was evaluated according to the Hirayabashi method (postoperative JOA score-preoperative JOA score)/(17–preoperative JOA score) × 100% [7,9]. An improvement rate of > 75% was considered excellent, 50% to 74% good, 25% to 49% fair, and 25% < poor. Furthermore, the achieved JOA score was calculated as the postoperative JOA score-preoperative JOA score [12]. We assessed ambulatory status with Nurick grade. We also measured postoperative axial pain on the visual analogue scale (VAS) 12 months after the initial surgery.

### 2.5. Radiological evaluation

**Figure 2:**
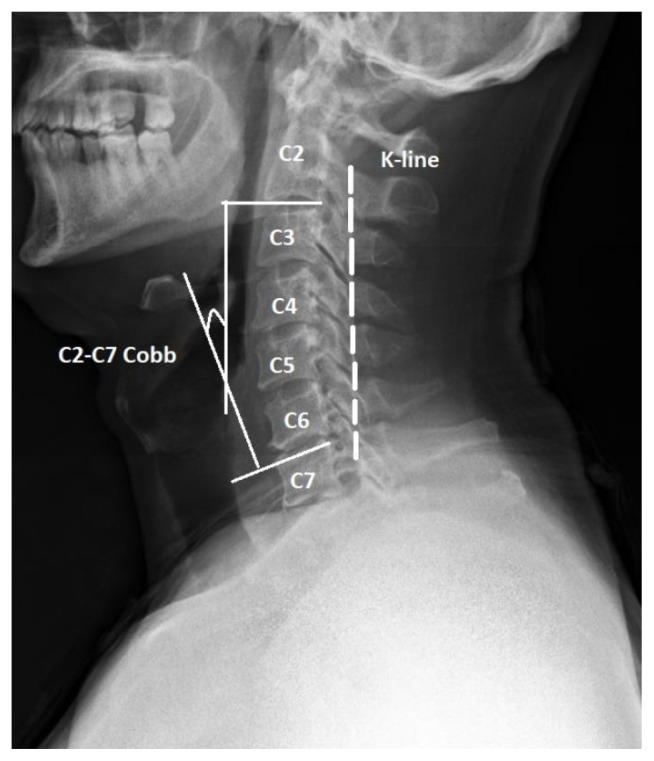
Lateralc-spine x-ray. We calculated both the Cobblor doticangle (LA) (angle between lower C2 end plate and superior C7 endplate) and the kyphosis line (K-line) (in the neutral position of the neck drawn between midpoints of the spinal canal C2 to C7).

**Figure 3A:**
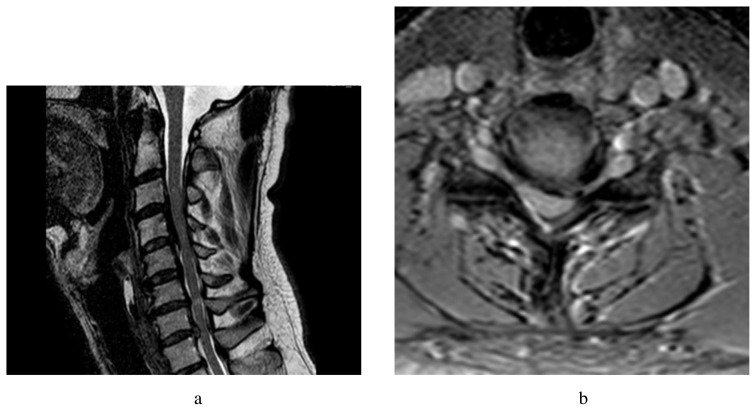
Postoperative sagittal T2 weighted MRI is demonstrating enlargement of the cervical spinal canal and relief of CSF passaged raw attention. **B**. Postoperative axial T2 weighted MRI is showing enlargement of the cervical spinal canal.

Lateral, anteroposterior, and dynamic (flexion and extension) radiographs of the cervical spine were obtained preoperatively, immediately after the operation and at the 12 month follow-up. Lateral X-rays were in a comfortable standing position. Cervical lordosis was measured by C2–C7 Cobb lordotic angle (LA), which was calculated by the inferior C2 endplate and superior C7 endplate in standing lateral X-ray and used for assessing the cervical alignment. The kyphosis line (K-line) in the neutral neck position (NNP), which is drawn from the center of the canal at C2 to the center of the canal at C7, is used for deciding on the surgical approach for patients with OPLL (Figure 2). The patients whose K-line (+) in the NNP underwent open-door laminoplasty (n = 11 patients). Computed tomography (CT) and magnetic resonance imaging (MRI) was performed in all patients preoperatively and postoperatively (Figures 3A and B). According to Yagi et al., the spinal cord’s signal intensity was assessed on MRI pre- and postoperatively on the T1 and T2 weighted slices. Grade I = high signal intensity change area on T2-weighted MR images limited to 1-disc level, with no hypointensity observed on T1-weighted sagittal MR images; Grade II = high signal intensity change area on T2-weighted MR images beyond 1 disc level, but with no hypointensity on T1-weighted sagittal MR images; Grade III = intramedullary hypointensity area observed on T1-weighted MR images in sagittal views [13].

### 2.6. Statistical analysis

We used IBM SPSS Statistics v. 17.0 software (IBM Corporation, Armonk, NY, USA) for data analysis. For continuous variables, normality was checked using the Shapiro–Wilk test, and the assumption of homogeneity of variances was tested with the Levene’s test. Descriptive statistics were expressed as mean ± SD for normally distributed continuous, median (range) for nonnormally distributed continuous, and percentage for categorical variables. Wilcoxon sign rank test and paired samples t-test were used to test differences between pre- and postoperative values of nonparametric and parametric variables, respectively. Spearman’s rank-order correlation coefficients were calculated to determine degrees of association between two variables. A p-value of less than 0.05 was accepted as statistically significant.

## 3. Results

**Figure 4:**
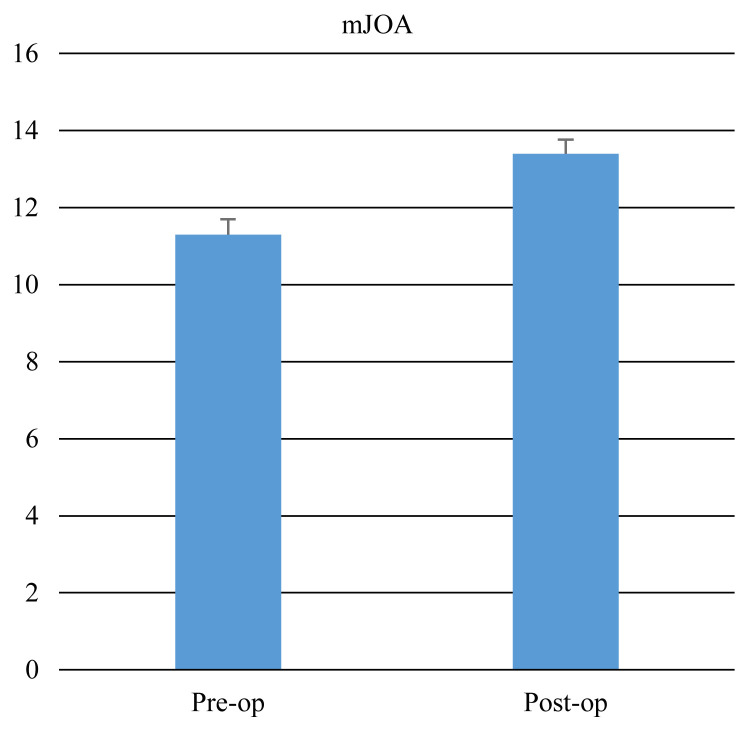
The patients’ median mJOA score increased from 11 (range 6–15) to 13.5 (range 9–16) postoperatively.

**Figure 5:**
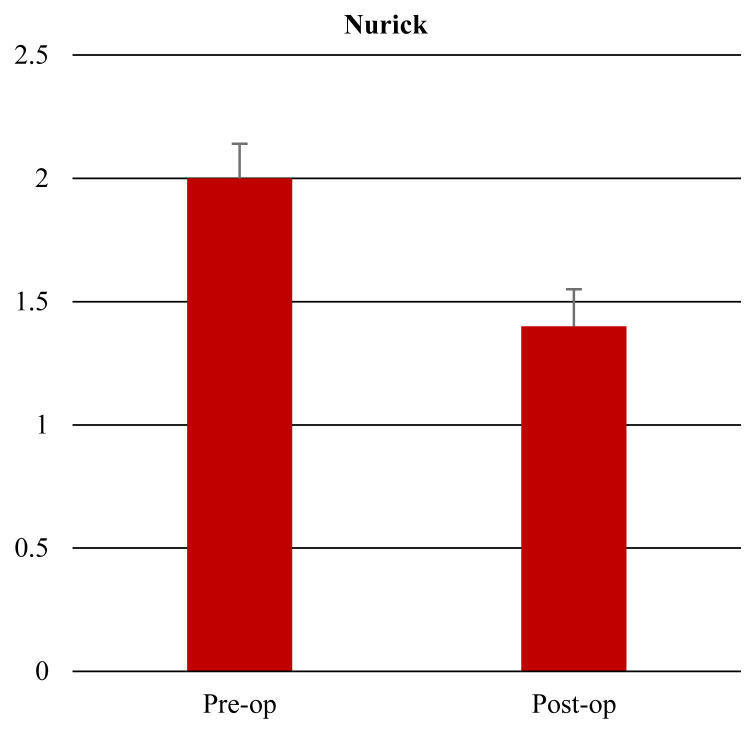
The patients› median Nurick grade decreased from 2 (range 1–3) to 1 (range 0–3) postoperatively.

Clinical and radiological characteristics of the patients including age, sex, duration of symptoms, duration of operation, pre- and postoperative VAS, mJOA scores, Nurickgrade, C2–C7 Cobb lordotic angle (LA), and type of ossification of the posterior longitudinal ligament (OPLL) are summarized in Table 1. The median duration of the symptoms was 12 months (range 2–24). The median duration of operation was 3 h (range 2–4). The preoperative and postoperative mJOA score, Nurick grade, VAS, and C2–7 Cobb angle values are presented in Table 2. The median mJOA score was 11 (range 6–15) preoperatively and 13.5 (range 9–16) postoperatively, with a mean recovery rate of 55% (range 0–75). The achieved JOA score was 1.5 (range 1–2). Patients’ mJOA score improvement was statistically significant (p < 0.001) (Figure 4). The median Nurick grade was 2 (range 1–3) preoperatively and 1 (range 0–3) postoperatively, with a statistically significant difference (p < 0.001) (Figure 5). We also represent the correlation between Nurick grade and mJOA score. The correlation of the improvement after surgery between the Nurick grade and the mJOA was statistically significant (p < 0.001) (Table 3). Twelve patients were older than 70 years old, with an average of 73 ± 0.8 years (range 70–80). This group of elderly patients has 11 ± 0.3 months (range 4–24 months) symptom duration. The correlation coefficients between age and duration of symptoms with differences in mJOA and Nurick grade are shown in Table 4. There was no statistically significant correlation between mJOA-Nurick grade-age and duration of symptoms (p > 0.05). Intramedullary signal intensity change was observed preoperatively in 27 (76.4 %) of 34 patients (5, 16, and 6 with grade I, grade II, and grade III, respectively). Postoperatively signal intensity improved in 7 patients (25.9%), worsened in 2 patients (7.4%), and remained unchanged in 18 patients (66.7%). The recovery rate was low in patients with Grade III intramedullary signal intensity (Table 5).

C2–C7 lordotic angle was 7.5 ° (range (−6) – (+39)) preoperatively and 12.5 ° (range (−2)–(+38)) postoperatively. The difference was statistically significant (p = 0.044). A total of 11 K-line (+) OPLL patients were enrolled to the study, and the mean mJOA score improved statistically significantly from 10.87 ± 1.70 to 13.29 ± 1.70 at the 12th-month follow-up (p < 0.001). The mean VAS of patientneck pain was 2.66 ± 1.4 preoperatively and 1.59 ± 1.4 postoperatively at 12 months with a statistically significant difference (p < 0.001). Postoperative complications were wound infection, cerebrospinal fluid leak, deep venous thrombosis, urinary tract infection, and C5 palsy. We did see neither perioperative deaths nor neurological worsening of the patients. Table 6 provides a summary of complications. All of the complications were successfully managed without any neurological deterioration.

## 4. Discussion

The incidence of CSM is increasing worldwide due to the prolonged lifespan [14]. Cervical spinal cord decompression is achieved by many different techniques, including anterior, posterior, or combined surgeries. Compared to the anterior approach, laminoplasty has fewer complication rates, is technically easier, and most surgeons prefer it if the patient has multi-level pathology [8,9]. In 1976, Hirabayashi described the open-door laminoplasty technique, a nonfusion alternative to removing multi-level spinal cord compression, has become a frequently applied surgical procedure for CSM patients. The aim of surgery is to maintain objective clinical improvement and adequate spinal cord decompression [7,11,15,16]. Recently, clinical studies in the literature have demonstrated that cervical laminoplasty has better clinical results than conventional laminectomy [5,17,18]. Many laminoplasty procedures exist including en bloc cervical laminoplasty with acrocristectomy, the use of hydroxyapatite spacers, allograft, autograft, titanium miniplates-screws, or sutures [4,15,19–21]. The techniques were successful in spinal cord decompression and very effective for treating cervical myelopathy. Tumturk et al. performed en bloc laminoplasty while protecting posterior structure with acrocristectomy at C6–7 level and concluded it as an effective technique for decompression, preserving spinal alignment, and postoperative axial pain [21]. Yang et al. showed that the laminoplasty procedure applied by fixing with suture provides a significant benefit in CSM patients and provides 1 Nurick score increase [22]. Stamates et al. performed open-door laminoplasty with autograft. They reported Nurick score improvement (2.25 ± 0.8 at 1-year follow-up), Neck disability index improvement (23.9 ± 10.9% at 1-year follow-up), and VAS improvement (1.78 ± 1.04 at 1-year follow-up) [20]. Yang et al. observed a 1.75 Nurick score increase after laminoplasty with miniplates-screws [18]. Similarly, in our study, in the preoperative period, we found the mean VAS neck pain of the patients to be 2.66 ± 1.4 preoperatively and 2.09 ± 1.4 postoperatively at 1-year follow-up (p < 0.001), and the median Nurick grade was found to be 2 (range 1–3) preoperatively and 1 (range 0–3) postoperatively at one year follow up (p < 0.001). Li et al. observed an improvement of 75 % in the mJOA score [19]. In the present study, we demonstrated similar improvement in the median mJOA score (11 preoperatively, and 13.5 postoperatively, with a 55% recovery rate). Nurick grade represents different functional abilities, such that it is used for ambulatory status of the patients, whereas JOA assesses upper limb and sensory function [23,24]. Vitztum et al. reported different CSM scores, and they showed that the Nurick and JOA scores of the patients were well correlated between the preoperative and postoperative terms [25]. Similar to previous studies, Nurick grades and mJOA scores of the patients in our study were highly correlated, too [3].

Surgical prognosis is affected by many conditions, such as the patient’s age, how severe the myelopathy is, and the signal intensity changes seen on MRI [10]. There is evidence in the literature that surgical outcomes of cervical laminoplasty are satisfactory in elderly patients [26,27]. In the meta-analysis performed by Takeshima et al., early intervention is recommended for advanced age CSM patients before these patients show neurological regression [28]. If the patient was older than 65, he had longer; if over 80 years old, he had a shorter symptom duration [26,29,30]. Notani et al. showed that the need for surgery and symptom onset was more straight forward in very elderly patients. The patients’ JOA scores were lower than the younger ones, and they concluded that there was a relationship between advanced age and spinal cord tenderness [14]. In our study, it is seen that there is no statistically significant relationship between mJOA score and age in patients older than 70 years. Furthermore, the JOA recovery rate was also lower in the elderly. This finding can be explained by a small number of patients and a longer duration of symptoms in our study. Similarly, in a previous study by Machino et al., the mJOA score calculation performed to evaluate postoperative recovery does not show a significant decrease in patients over 70 years of age. When these results are evaluated, it is thought that laminoplasty can be applied as a useful and beneficial procedure in elderly patients with CSM [31]. Early surgical intervention as soon as possible will improve postoperative outcome who had lower mJOA scores. The patients’ functional results deteriorate with the spinal cord intensity changes seen on MRI [32]. The hypointense area on T1-weighted MRI is thought to be the most noticeable deterioration indicator in CSM patient outcomes [2,24]. Both low signal intensityon T1 and high intensity on T2-weighted images are considered as an indicator of irreversible damage to the spinal cord [31,33]. The present study shows that grade III signal intensity changes were 21% (6/34), mostly seen in the elderly patient group. These patients showed less improvement than patients with grade I and II intensity changes. The K-line in NNP, which calculates the cervical alignment and OPLL thickness, is seen as a practical parameter that can be easily evaluated [34,35]. Among the patients who underwent laminoplasty, patients with K-line (−) OPLL and those with high OPLL thickness (OPLL covering more than 60% of the spinal canal diameter) constitute the group with poor patient outcomes [34,36].

However, considering the JOA score, neck pain, recovery after laminoplasty, and Nurick grade, some studies show that cervical kyphosis has no significant effect on patient outcomes. When patients with increased cervical kyphosis and lordosis were evaluated, it was shown that the surgical results of these patients were not considerably affected after the laminoplasty procedure [9,12]. In the present study, we performed open-door laminoplasty only for K-line (+) OPLL patients with an excellent clinical outcome postoperatively. Our opinion is that postoperative cervical kyphosis causes deterioration in spinal cord function, increases cervical spinal instability, and poses the main problem in K-line (−) OPLL patients. Machino et al. enrolled 520 consecutive CSM patients with a mean follow-up period of 33.3 months and reported the mean cervical lordotic angle in the NNP increased from 11.9 ° preoperatively to 13.6 ° postoperatively [31]. Similarly, in our study, it is seen that the cervical lordotic angle at the neutral neck position increased significantly from 10.37 to 12.72 at 12-month follow-up due to the preservation of the posterior tension band between C2–C7. Yang et al. reported a 1.74 improvement in neck pain VAS after open-door laminoplasty using miniplate and screws [18]. In some studies, postoperative axial neck pain worsened after laminoplasty, and the authors suggested unilateral dissection or muscle-preserving surgery [37,38]. In our study, it is seen that there is an increase of 1.07 points when the VAS values of the patients are calculated. This result may be relevant to proper patient selection and to keep patients in a cervical collar for at least 3 weeks.

Although the patient number in our study is comparable to other studies in the literature, it has a small sample size. CSM is a chronic disease, and the benefit of cervical laminoplasty and stability of the treatment should be followed up longer than 12 months. The retrospective design of the study is limiting to reach out to patients.

## 5. Conclusion

Open door laminoplasty with titanium miniplate and screws is a good and reliable surgical technique to treat cervical spondylotic myelopathy. Our present findings highlight that laminoplasty for CSM can limit the progression of symptoms, improve prognosis, and change myelopathy’s natural progression. Long-term results are necessary to support this conclusion further.

## Figures and Tables

**Table 1:** Baseline demographic, and clinical characteristics of cases.

	n = 34
Age (years)	63.7 ± 10.3
Range of ages (years)	33–80
Sex	
Male	24 (70.6%)
Female	10 (29.4%)
Duration of operation (h)	3 (2–4)
Duration of symptoms (months)	12 (2–24)
Type of OPLL	
Continue	3 (27.4%)
Segmental	4 (36.3%)
Mixed	4 (36.3%)
Improvement (%)	40 (0–75)
Achieved JOA	1.5 (1–2)

The values are presented as mean ± SD, median (min–max). JOA: Japanese Orthopaedic Association; OPLL: Ossification of the posterior longitudinal ligament.

**Table 2:** Summary of the pre and postoperative mJOA score, Nurick grade, VAS, and C2–7 Cobb angle values.

	Preoperative	Postoperative	p-value	Difference
mJOA score	11 (6–15)	13.5 (9–16)	<0.001†	2 (0–6)
Nurick grade	2 (1–3)	1 (0–3)	<0.001†	0 (−2–0)
VAS	2.66 ± 1.7	1.59 ± 1.4	<0.05‡	1.07 ± 0.4
C2–7 Cobb angle (°)	7.5* ((−6)–(+39))	12.5* ((−2)–(+38))	0.044†	5* (0–9)

† Wilcoxon sign rank test,

‡ Paired t-test.

The values are presented as mean ± SD, median (min–max).

mJOA: Modified Japanese Orthopaedic Association; VAS: Visual analogue scale.

**Table 3:** The results of correlation analysis among mJOA score and Nurick grade.

	Preoperative	Postoperative	Difference
mJOA score – Nurick grade			
Coefficient of correlation	−0.946	−0.818	−0.485
p-value †	<0.001	<0.001	0.004

† Spearman’s rank-order correlation analysis.

mJOA: Modified Japanese Orthopaedic Association.

**Table 4:** The correlation coefficients and statistically significance levels between age, and duration of symptoms with the differences in mJOA score, Nurick grade, and C2–7 Cobb angle depending on operation.

	mJOA score	Nurick grade	C2–7 Cobb angle
Age			
Coefficient of correlation	−0.328	0.242	0.070
p-value †	0.059	0.167	0.705
Duration of symptoms			
Coefficient of correlation	0.214	−0.210	0.092
p-value †	0.224	0.234	0.615

† Spearman’s rank-order correlation analysis.

mJOA: Modified Japanese Orthopaedic Association.

**Table 5:** Preoperative and postoperative MRI grades of the patients.

	Number (%) of the patients			
	Preoperative	Postoperative		
		Unchanged	Improved	Worsened
No signal change	7 (21.7)	7 (100)	0 (0)	0 (0)
*Grade I	5 (14.2)	2 (40)	3 (60)	0 (0)
*Grade II	16 (44.8)	10 (62.5)	4 (25)	2 (12.5)
*Grade III	6 (19.3)	6 (100)	0 (0)	0 (0)

The values are presented as n (%).

* Grade I = high signal intensity change area on T2-weighted MR images limited to 1-disc level, with no hypointensity observed on T1-weighted sagittal MR images; Grade II = high signal intensity change area on T2-weighted MR images beyond 1 disc level, but with no hypointensity on T1-weighted sagittal MR images; Grade III = intramedullary hypointensity area observed on T1-weighted MR images in sagittal views [39].

MRI: Magnetic resonance imaging.

**Table 6:** Perioperative complications.

Complication	Number (%) of patients
Wound infection	5 (14.7)
Cerebrospinal fluid leak	2 (5.9)
Deep venous thrombosis	2 (5.9)
Urinary tract infection	3 (8.8)
C5 palsy	3 (8.8)

The values are presented as n (%).
